# The Impact of Green FinTech Promote Corporate Carbon Neutrality: Evidence from the Perspective of Financing Incentives and Scale Quality

**DOI:** 10.3390/e28010006

**Published:** 2025-12-20

**Authors:** Lei Zhuang, Chuang Wu

**Affiliations:** School of Economics and Management, Nanjing Tech University, Nanjing 211816, China

**Keywords:** fintech, green finance, carbon neutrality, ESG, financing incentives, enterprise scale

## Abstract

As an in-depth integration of green capital chains and technological innovation chains, green fintech provides strong support for enterprises in promoting green and low-carbon development and achieving carbon neutrality. Based on relevant data from Chinese listed companies between 2014 and 2023, this study constructs indices for green fintech development and corporate carbon neutrality to empirically examine the impact of green fintech on corporate carbon neutrality. Benchmark regression results show that green fintech exerts a significantly positive effect on corporate carbon neutrality. A mediation analysis of financing incentives indicates that alleviating corporate financing constraints and reducing financial distress are effective pathways through which green fintech facilitates carbon neutrality. Furthermore, a moderating effect analysis reveals that green fintech plays a more pronounced role in enhancing carbon neutrality for enterprises with higher audit quality and larger operational scales. Accordingly, policy recommendations are proposed, focusing on establishing a green fintech service-sharing platform, providing targeted policy support, and improving carbon information disclosure mechanisms.

## 1. Introduction

Governments and international organizations are setting ambitious carbon neutrality targets, such as achieving net-zero emissions by 2060. In this context, corporations face growing pressure to reduce their carbon footprint, and financial mechanisms are increasingly critical to realizing these goals. Recent research has shown substantial interest in green fintech, which merges financial technology with sustainability objectives to enable innovative solutions like green bonds, carbon trading platforms, and ESG-focused digital finance (Shen et al., 2025) [1]. Enterprises encounter significant challenges in pursuing carbon neutrality. The development cycle for carbon-neutral technologies is often lengthy and capital-intensive, yet traditional financial instruments provide limited support for green innovation. Financial institutions, moreover, frequently lack the capacity to accurately assess the risks of low-carbon projects. Policy frameworks and market mechanisms remain underdeveloped, and many companies lack sustained, stable incentives to reduce emissions. Green fintech—integrating finance with digital technology—addresses these gaps by leveraging advanced tools such as big data, blockchain, and artificial intelligence. These technologies optimize resource allocation, lower transaction costs, and improve risk management (Puschmann & Khmarskyi, 2024) [2]. They play a pivotal role in meeting enterprises’ innovation financing needs and in identifying and mitigating risks within low-carbon initiatives. In 2023, China’s central government highlighted “technology finance and green finance” as one of five strategic priorities. Local governments have since introduced measures to expand carbon emission reduction support tools and have required financial institutions to enhance carbon benefit assessments. Cities like Shanghai and Shenzhen have piloted “carbon account” models that directly link corporate carbon performance to credit ratings and financing costs—creating tangible incentives for proactive emission reductions. Therefore, leveraging green fintech to empower enterprises in achieving carbon neutrality and accelerating the shift toward a green, low-carbon development model has become a crucial element of effective carbon neutrality strategy implementation (Wan et al., 2025) [3].

Regarding existing research on corporate carbon neutrality, technological approaches predominantly focus on achieving a structural balance between carbon emissions and absorption through carbon substitution, reduction, capture, and storage. Specifically, carbon substitution entails replacing fossil fuels with renewable energy sources such as wind, hydro, and solar power, as well as advancing hydrogen energy technologies, thereby facilitating the decarbonization of energy supply (Zou et al., 2021) [4]. Carbon reduction, meanwhile, centers on lowering carbon emissions without eliminating the carbon source itself. This may include the development of new materials that reduce the embodied carbon in products (Zhao et al., 2023) [5]. Carbon capture technologies encompass biological, physical, and chemical absorption. Biological methods rely on photosynthesis to fix atmospheric carbon dioxide in vegetation, while physical absorption exploits the dissolution properties of CO_2_ under low temperature and high pressure. Chemical absorption utilizes solvents to isolate and convert carbon dioxide into reusable chemicals or fuels, such as methanol (Huang & Luo, 2025) [6]. Carbon sequestration refers to the transportation and long-term storage of captured CO_2_ in specific geological formations, such as depleted oil and gas reservoirs, deep saline aquifers with strong containment properties, or deep-sea “carbon lakes” (Leung et al., 2014) [7]. In terms of measurement methodologies, two main approaches have been adopted in the literature to assess corporate carbon neutrality. One is the indicator substitution method, which uses proxy variables—such as corporate green innovation (Chang, 2011) [8] or ESG performance (Qian, 2024) [9]—to reflect the degree of corporate green transformation. The other involves constructing a composite indicator system. For instance, some studies evaluate green development based on five dimensions: green systems, operations, organizational DNA, social responsibility, and investment, with aggregated sub-indicator scores forming a composite index (Orazalin & Mahmood, 2021) [10]. Others design systems covering economic, social, and ecological performance, applying methods like the analytic hierarchy process or entropy weighting to assign indicator importance (Wang et al., 2025) [11]. There are also frameworks incorporating corporate green culture, strategy, innovation, investment, production, and emissions, where weights are determined through analytic hierarchy processes combined with BP neural networks (Yu et al., 2024) [12].

When it comes to the role of finance in driving technological innovation and green development, research increasingly highlights green fintech as a pivotal enabler. By integrating technological innovation with financial capital through multi-stakeholder collaboration, green fintech provides crucial funding support for green technological activities, thereby advancing carbon neutrality innovation and corporate green transformation. Spatial econometric models reveal that green fintech effectively promotes industrial green transformation with notable spillover effects (Ge et al., 2022) [13]. Difference-in-differences analyses further confirm that green fintech policies significantly stimulate urban green innovation, with effects intensifying over time (Liu et al., 2023) [14]. These policies are also found to meaningfully inhibit environmental pollution, again exhibiting spatial spillover characteristics (Udeagha & Muchapondwa, 2023) [15]. Quasi-natural experiments indicate that green fintech enhances urban green innovation through improved resource allocation efficiency and talent aggregation. Studies focusing on eastern coastal urban agglomerations underline the pivotal role of the green fintech ecosystem in spurring green innovation (Ma et al., 2025) [16]. Dual machine learning approaches also confirm a significant positive impact of green fintech on low-carbon technological innovation (Shah et al., 2025) [17]. Research based on EU dynamic panel data demonstrates that venture capital positively influences patenting activities and facilitates corporate green transition (Faria & Barbosa, 2014) [18]. Studies in the U.S. and EU contexts likewise highlight the important role of R&D expenditure in reducing carbon dioxide emissions (Fernández et al., 2018) [19].

In constructing green fintech indicator systems, scholars often draw from ecological theory to conceptualize a “community subsystem” composed of governmental bodies, technology-innovating enterprises, and green fintech markets, alongside an “environmental subsystem” encompassing foundational support and technological resource contexts (Zhong et al., 2023) [20]. Common approaches include characterizing green fintech development across five dimensions: science and technology finance, taxation and credit, venture capital, tech capital markets and insurance, green fintech environment, and green fintech output (Lv et al., 2021) [21]. Some studies select indicators across four dimensions—fintech resources, funding, output, and loans—applying arithmetic mean weighting (Wang et al., 2021) [22]. A more simplified method involves standardizing and summing four key financial variables—government S&T expenditure, S&T loan volume, venture capital investment, and tech capital market financing—to derive a Green Fintech Index (Wu et al., 2025) [23]. Many scholars employ the entropy method for weight determination, distinguishing between public and market-based green fintech, with the latter further categorized into commercial banks, technology capital markets, and venture capital segments (Cheng et al., 2023) [24]. Another perspective classifies indicators based on financing supply (by instrument type) and demand (by sector and unit), constructing a green fintech index through sequential aggregation of financing flows (Sun et al., 2021) [25]. Overall, the literature converges on the view that green fintech alleviates corporate financing constraints, thereby stimulating green innovation and accelerating low-carbon transformation (Li et al., 2025) [26].

The primary contribution of this study is to extend existing research by investigating, from a micro-level perspective, how green fintech influences corporate carbon neutrality. By constructing an enterprise carbon neutrality development index and a green fintech index—incorporating dimensions such as carbon substitution and reduction—this research empirically examines the impact of green fintech on corporate carbon performance. This study offers several key theoretical contributions: First, drawing on innovation economics and principal-agent theory, the research develops a green fintech development index and a corporate carbon neutrality index, incorporating dimensions of scale and quality (effectiveness and rigor). Utilizing a sample of Chinese A-share listed firms from 2014 to 2023, it provides a theoretically grounded micro-level analysis. Second, the study advances the sustainability and financial technology literature by explicitly linking digital finance instruments—such as green bonds and carbon trading platforms—to measurable corporate carbon neutrality outcomes. It offers a novel framework for understanding how financial innovation drives environmental performance, addressing a gap in prior research. Third, by analyzing how financing incentives and constraints shape corporate decision-making toward carbon neutrality, the research introduces a behavioral finance perspective into green finance, enriching the understanding of corporate environmental strategy. A conceptual model is developed that integrates technological innovation, financial mechanisms, and environmental goals. This bridges three previously siloed research streams, providing a more holistic view of the sustainability transition. Finally, the study connects financial accessibility not only to firm-level innovation capability but also to ecological network effects, highlighting the systemic role of green fintech in enabling collaborative and scalable climate action. Based on these empirical insights, targeted policy recommendations are proposed to support the implementation of corporate carbon neutrality via green fintech tools.

## 2. Theoretical Analysis and Hypotheses

### 2.1. The Direct Effect of Green FinTech in Promoting Carbon Neutrality for Enterprises

Under the “dual carbon” goals, it is imperative for enterprises to develop new products and processes through carbon-neutral technology research and development to achieve carbon balance (Wang et al., 2018) [27]. According to the theory of innovation economics, the new products and improved processes resulting from technological innovation drive industrial structure upgrading. Within this context, enterprises, universities, financial institutions, and other stakeholders form a green innovation ecosystem through knowledge spillover, collaborative R&D, and capital investment.

Green fintech not only directly funds corporate carbon-neutral technology projects but also engages in the entire project lifecycle—conducting pre-project evaluations and screenings, supervising and tracking innovation progress during project implementation, and performing output assessments and fund reviews post-project (Han et al., 2024) [28]. This comprehensive involvement helps minimize the adverse effects of R&D fund misallocation caused by principal–agent problems on corporate carbon neutrality efforts (Wang, 2018) [29]. Green fintech also supports high-quality economic growth. Economic development, along with improved living and employment conditions, attracts talent aggregation, thereby providing a solid human and financial resource foundation for enterprises to achieve carbon neutrality. Scientific and technological human resources are crucial for innovation, and talent concentration significantly enhances both corporate and regional innovation capabilities (De Winne & Sels, 2010) [30].

Financial institutions such as banks leverage the authority granted by debt contracts throughout the green fintech lifecycle to review corporate operational strategies, mitigate information asymmetry, improve resource allocation efficiency, and—through data-driven resource allocation mechanisms—accurately identify growth-oriented enterprises and high-quality projects. This facilitates the prioritized development of low-carbon industries such as digital and high-tech sectors, curbs the expansion of traditional energy-intensive and high-pollution industries, promotes industrial structure upgrading, and reduces energy consumption and pollutant emissions at the source.

Enterprises that attract green fintech resources send positive signals to the market regarding their strong environmental responsibility, making it easier to attract investors and partners. This not only lowers financing costs but also enhances corporate reputation and improves financing efficiency. Such a reputational effect can incentivize other firms to improve carbon information disclosure, strengthen environmental awareness, increase R&D investment in carbon offsetting, actively adopt zero-carbon and low-carbon technologies or smart energy management systems, boost the output of new green products, accelerate the application of green processes, and ultimately foster a virtuous cycle in the green industrial chain (Li, 2024) [31].

**Figure 1:**
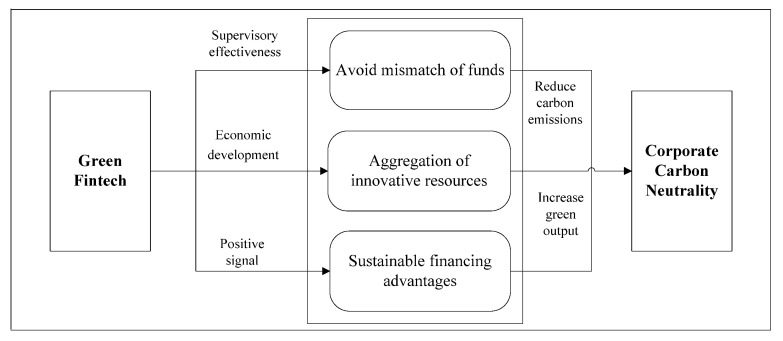
The logical relationship of green fintech promotes corporate carbon neutrality.

Therefore, green fintech promotes the efficient allocation of financial resources, leading to increased green output and reduced carbon emissions for enterprises, thereby exerting a positive impact on corporate carbon neutrality, as illustrated in Figure 1. Based on the above analysis, the following hypothesis is proposed:

**H1.**  *Green fintech has a positive impact on enterprises’ achievement of carbon neutrality.*

### 2.2. Intermediary Effect of Financing Incentives

Financing incentives serve as a crucial pathway through which green fintech supports enterprises in achieving carbon neutrality. Information asymmetry often leads external investors to worry that principal–agent problems may result in the misuse of funds, prompting them to approach corporate financing needs with caution. This manifests in higher risk premiums or even outright refusals to provide funding, which restricts external financing channels and creates financing constraints for enterprises. When facing such constraints, companies tend to adopt more conservative business strategies, prioritizing short-term survival by directing cash flow toward daily operations rather than long-term investments. This often leads to reduced capital allocation for carbon reduction technology R&D and green infrastructure projects, thereby delaying progress toward carbon neutrality. The core function of green fintech lies in providing targeted financial support during enterprises’ technology R&D and incubation phases (Metawa et al., 2022) [32]. The development of the financial industry has been shown to effectively ease corporate financing constraints and advance carbon-neutral development (Wang & Chen, 2023) [33]. Green fintech, through collaborative efforts between local governments and private investors, helps establish science and technology innovation funds that pool social financial resources (Yang & Wang, 2022) [34], thereby providing substantial funding for corporate low-carbon transformation.

Moreover, green fintech enables innovative enterprises to convert intellectual property into tangible assets through financial instruments such as intellectual property pledge financing, using patents and trademarks as collateral (Kwong et al., 2023) [35]. By leveraging big data analytics, green fintech can rapidly collect and analyze corporate operational and credit data. Even for small and medium-sized enterprises with limited credit histories, it can develop comprehensive credit profiles using transaction and supply chain data. This reduces information asymmetry and lowers information acquisition costs for investors, effectively expanding enterprises’ access to financing. Once financing constraints are alleviated, companies become more inclined to pursue social responsibilities, invest in green innovation projects with long-term returns, and actively advance their internal carbon neutrality initiatives. Based on this reasoning, the following hypothesis 2 is proposed:

**H2.**  *Green fintech has a positive impact on enterprises’ carbon neutrality through financing constraint adjustments.*

Green financial technology drives innovation in financial products and financing models, offering flexible financial support for corporate R&D projects. This effectively alleviates corporate financing constraints while enabling comprehensive oversight of R&D initiatives, thereby mitigating the misallocation of R&D funds due to principal–agent issues and ensuring efficient capital utilization. Addressing financial distress represents a survival instinct for enterprises seeking to avoid bankruptcy (Milne, 2014) [36]. Companies in financial difficulty must rapidly enhance profitability and implement strategies for management, operational, asset, and financial restructuring (Sudarsanam & Lai, 2001) [37] to consolidate high-quality assets and overcome financial challenges. Risk-averse managers, especially those with experience of financial hardship, tend to maintain high levels of liquid financial assets (Liu et al., 2024) [38] to increase their distance to financial default and avoid falling into distress.

However, an excessive focus on maintaining distance from financial default may come at the cost of reduced investment in low-carbon technologies and green production. While such a conservative financial strategy lowers default risk, it may also result in significant idle capital, undermining long-term growth and competitiveness. Green fintech enables continuous monitoring of corporate capital flows and debt repayment capabilities. By analyzing key indicators such as cash flow and debt-to-asset ratios, it dynamically assesses financial risks and automatically issues early warnings when anomalies are detected, helping firms avert financial crises. Furthermore, green fintech disperses risks associated with R&D projects, enhances corporate resilience, and allows companies to fully leverage financial resources for innovation while appropriately moderating their distance to financial default. This balanced approach helps prevent both resource idleness and severe financial distress. Based on this analysis, the following hypothesis 3 is proposed:

**H3.**  *Green fintech has a positive impact on corporate carbon neutrality by reducing the distance to financial default.*

### 2.3. Moderating Effect of Enterprise Scale and Quality

The scale and quality of enterprises are key factors influencing the effectiveness of green fintech in supporting corporate carbon neutrality. Currently, insufficient transparency in corporate carbon emissions data and uneven quality of carbon information disclosure make it challenging for investors and financial institutions to accurately assess companies’ progress toward carbon neutrality. External senior auditors, as independent third parties, often serve as informational intermediaries between firms and investors in capital markets (Watts & Zimmerman, 1983) [39]. By issuing professional audit reports, they enhance the transparency of corporate financial data and credit records. According to signaling theory, companies with high audit quality can convey positive signals to the market, differentiate themselves from peers, strengthen investor confidence, attract more innovative resources, and secure diversified financing support—including higher-quality ESG disclosures.

Advancing corporate carbon neutrality generates significant positive externalities. However, there is often a divergence of interests between corporate managers and actual controllers, which may lead firms to prioritize short-term economic gains over long-term social responsibilities, thereby impeding the effective implementation of carbon neutrality goals. External auditing, independent of internal corporate controls, is more likely to uncover concealed operational issues and curb potential greenwashing by management (Feng et al., 2024) [40]. This encourages companies to internalize the positive externalities of carbon neutrality, uphold their environmental responsibilities, and implement green fintech resources in a substantive manner. Based on this analysis, the following hypothesis 4 is proposed:

**H4.**  *Green fintech plays a more significant role in promoting carbon neutrality for enterprises with high audit quality.*

The long payback periods and high investment costs associated with carbon neutrality technologies, compounded by the elevated risks inherent in complex innovation processes, often lead business operators to adopt a highly cautious approach toward such R&D initiatives. In contrast, large enterprises typically possess a stronger financial foundation. Their diversified operations help effectively disperse R&D risks (Karlsson & Olsson, 1998) [41], and their established credit records facilitate access to lower-cost financing. Moreover, large enterprises tend to exhibit greater willingness to engage in R&D (Fu, 2024) [42]. These advantages enable large firms to demonstrate both the confidence and capacity to establish dedicated R&D teams for carbon reduction projects, commit to substantial investments, and leverage green fintech resources to drive innovation and breakthroughs in carbon neutrality-related technologies. Consequently, they can accelerate the renewal of green infrastructure and the transition toward greener production processes.

Large-scale enterprises also wield greater bargaining power, placing them in a stronger position during price negotiations for low-carbon technology equipment or clean energy procurement. This reduces the costs associated with green transformation and further enhances their incentive to invest in carbon-neutral technology R&D. Additionally, while large enterprises attract greater attention, they also face heightened expectations and pressure from a wide range of stakeholders—including governments, society, and investors. Such pressures encourage them to prioritize social responsibility (ESG) and, in turn, motivate more proactive utilization of green fintech resources to advance carbon neutrality goals. Innovative activities often achieve optimal production efficiency within large enterprises, which are generally early adopters of cutting-edge technologies and innovations. By deploying green fintech resources, they can rapidly implement technological advancements and reduce promotion costs through their demonstration effect within the industry. Research indicates that large-scale enterprises are able to achieve innovation outcomes more swiftly (Dokas et al., 2023) [43]. Based on this analysis, the following hypothesis 5 is proposed:

**H5.**  *The promoting effect of green fintech on carbon neutrality is more significant for large-scale enterprises.*

## 3. Methods and Data

### 3.1. Variable Selection

#### 3.1.1. Explained Variable

The explained variable is the Enterprise Carbon Neutrality Development Index (*Ecn*). To measure the level of carbon neutrality development in enterprises, an indicator system was constructed based on two main dimensions: carbon offsetting and carbon reduction. From the carbon offsetting perspective, four secondary indicators were selected: environmental management system, reduction concept, green innovation input, and green innovation output. From the carbon reduction perspective, three secondary indicators were chosen: enterprise carbon emissions, environmental accidents, and carbon information disclosure. A total of 20 tertiary indicators were identified to form the Enterprise Carbon Neutrality Development Index system, as detailed in Table A1.

The data for each indicator were sourced from the CSMAR database and the China Research Data Service Platform. Considering data availability, the study selected A-share listed companies on the Shanghai and Shenzhen stock exchanges from 2014 to 2023 as the research sample. The sample was screened as follows: ST and PT companies, financial firms, insolvent companies, companies listed after 2022, and those with missing key variables were excluded. After these steps, a total of 4053 listed companies were retained, yielding 30,653 valid observations.

To measure the emission reduction concept, a score was assigned based on corporate disclosures. Specifically, one point was given for each of the following: environmental protection concepts, goals, management systems, education and training programs, special initiatives, awards or honors, emergency response mechanisms for environmental incidents, and the implementation of the “three simultaneous” system. The absence of any of these items received a score of 0. The total points constituted the score for the emission reduction concept.

Regarding enterprise carbon emissions, due to the limited availability of publicly disclosed data, the estimation method followed existing literature (Lin, 2025) [44], in which corporate carbon emissions were approximated based on industry-level emission data. The calculation formula is as follows:

(1) $$CEP\;=\frac{OI}{\frac{ICE}{IBC}+1}\times OC$$

where *CEP* represents enterprise carbon emission performance, *OI* represents enterprise operating income, *OC* represents enterprise operating cost, *ICE* represents industry carbon emissions, *IBC* represents industry main business cost.

(2) $$CDE=\frac{ICE}{IBC}\times OC$$

Among them, *CDE* represents enterprise carbon dioxide emissions.

Regarding environmental accidents, a score of 1 is assigned for each disclosed incident involving key pollution monitoring units, sudden environmental accidents, environmental violations, or petition cases; otherwise, a score of 0 is given. The sum of these scores constitutes the environmental accident score. For carbon information disclosure, emissions of wastewater, COD, SO_2_, CO_2_, smoke dust, and dust, as well as the generation of industrial solid waste, are scored as follows: 0 for no disclosure, 1 for qualitative disclosure, and 2 for quantitative disclosure. The total score reflects the level of environmental liability disclosure. Similarly, for the reduction and treatment of waste gas, wastewater, dust and smoke dust, solid waste, noise, and light pollution, along with the implementation of clean production, the scoring follows the same pattern: 0 for no disclosure, 1 for qualitative disclosure, and 2 for quantitative disclosure. The aggregate score represents the environmental governance disclosure performance. Due to space constraints, the detailed calculation results of the enterprise carbon neutrality development index are not presented here.

As illustrated in Table 1, the overall enterprise carbon neutrality development index in China has shown a steady upward trend. Its average value increased from 22.56 in 2014 to 32.36 in 2023. The range and standard deviation of the index have also gradually widened, with the standard deviation rising from 8.87 in 2014 to 11.48 in 2023. This indicates that while the overall carbon neutrality development level of listed companies in China is improving, the disparity in carbon neutrality performance among enterprises is also expanding.

#### 3.1.2. Explanatory Variable

The core explanatory variable is the Green Fintech Development Index (*GTF*). Based on four sustainable dimensions—resources, funding, financing, and output—twelve secondary indicators are selected, including R&D human resources, R&D institutions, financial resources, intensity of fiscal allocation, intensity of R&D investment, scale of R&D investment, intensity of financial support, intensity of venture capital, scale of market financing, technology market transaction rate, paper output rate, and new product output rate. The indicator system for the Green Fintech Development Index is presented in Table A2. Data for the indicators are primarily sourced from the National Bureau of Statistics, the EPS database, and the China Statistical Yearbook on Science and Technology.

The entropy method was applied to calculate the Green Fintech Development Index for each Chinese province from 2014 to 2023. As shown in Table 2, the overall development of green fintech in China has been trending upward. The standard deviation across provinces increased from 6.75 in 2014 to 10.87 in 2023, indicating a considerable regional disparity in the level of green fintech development.

#### 3.1.3. Mediating Variables

The mediating variables for financing incentives are financial default distance (*FZ*) and financing constraints (*SA*). Drawing on relevant research (Mushafiq et al., 2024) [45], the formula for calculating the financial default distance variable is given in Equation (3), where *X*_1_ is the ratio of working capital to total assets, *X*_2_ is the ratio of retained earnings to total assets, *X*_3_ is the ratio of equity market value to total liabilities, *X*_4_ is the ratio of operating income to total assets, and *X*_5_ is the ratio of earnings before interest and taxes (*EBIT*) to total assets. A larger *FZ* value indicates that the enterprise is further from financial default, implying a lower risk of default. Financing constraints are measured based on methods from relevant literature (Jabbouri et al., 2024) [46], using firm size and firm age. The calculation formula is shown in Equation (4), where size represents firm scale and age represents firm age. The calculated SA values are typically negative; the closer the SA value is to 0, the higher the financing constraints faced by the enterprise.

(3) $$FZ=1.2X_{1}+1.4X_{2}+0.6X_{3}+0.999X_{4}+3.3X_{5}$$

(4) $$SA=-0.737size+0.043{size}^{2}-0.04age$$

The moderating variables for scale-quality are audit quality and enterprise size (*ES*). Both moderating variables are binary, taking values of 0 or 1. For audit quality, a value of 1 is assigned if the enterprise’s auditor in the current year is one of the Big Four international accounting firms or one of the top ten domestic audit firms ranked by evaluation score or revenue; otherwise, the value is 0. For enterprise size, firms are categorized into large-scale and small-scale based on the median of total assets (*Size*). Firms with total assets above the median are assigned a value of 1, while those below or equal to the median are assigned a value of 0.

#### 3.1.4. Control Variables

This article selects the following control variables: Enterprise Age (*Age*), return on assets (*ROA*), total asset growth rate (*TAGR*), and equity Balance (*Balance*). While controlling for both individual and time-invariant factors, the descriptive statistics of each variable are shown in Table 3.

### 3.2. Model Construction

Taking the Enterprise Carbon Neutrality Development Index (*Ecn*) as the explained variable and the Green financial Technology development Index (*TF*) as the explained variable, and selecting enterprise Age (*Age*), return on assets (*ROA*), total asset growth rate (*TAGR*), and equity Balance (*Balance*) as control variables, a bidirectional fixed effects model was constructed for empirical analysis. The model Settings are as follows:

(5) $${Ecn}_{it}=\alpha_{0}+\alpha_{1}{GTF}_{it}+\alpha_{2}CV\;+\mu_{i}+\lambda_{t}+\varepsilon_{it}$$

According to the mediating effect method, financing constraints and financial default distance are selected as mediating variables, and the model is set as follows:

(6) $$M_{it}={\;\alpha }_{0}+\alpha_{1}G{TF}_{it}+\alpha_{2}CV\;+{\;\mu }_{i}+{\;\lambda }_{t}+\varepsilon_{it}$$

Considering the moderating effects of enterprise audit quality and enterprise scale, a moderating effect model is constructed for verification. The model is set as follows:

(7) $${Ecn}_{it}=\alpha_{0}+\alpha_{1}G{TF}_{it}+{\;\beta }_{1}{TF}_{it}\times R_{it}{+{\;\beta }_{2}R_{it}+\alpha }_{2}CV\;+\mu_{i}+\lambda_{t}+{\;\varepsilon }_{it}$$

Among them, ${Ecn}_{it}$ represents the explained variable enterprise carbon neutrality development index of enterprise *i* during period *t*, ${GTF}_{it}$ represents the core explanatory variable green financial technology development index of enterprise *i* during period *t*, CV is a series of control variables, and $M_{it}$ is the mediating variable, including financing constraint *SA* and financial default distance *FZ*. $R_{it}$ is the adjusting variable, including Audit quality and enterprise scale *ES*, *α*_0_ is the constant term, *µ* controls the individual fixed effect, *λ* controls the time fixed effect, and *ε* is the random disturbance term.

## 4. Empirical Analysis

### 4.1. Benchmark Regression

The bidirectional fixed-effects model is employed to examine the impact of green fintech on corporate carbon neutrality. The baseline regression results are reported in Table 4. Column (1) presents the regression without control variables, in which the coefficient of the core explanatory variable, green fintech, is significantly positive at the 1% level, indicating that green fintech promotes corporate carbon neutrality development. After including control variables and accounting for individual and time fixed effects, column (2) reports the benchmark regression results. These results demonstrate that even after controlling for firm age, return on assets, total asset growth rate, and equity balance, the positive effect of green fintech on corporate carbon neutrality remains significant at the 1% level. Specifically, a 1% increase in the green fintech development index is associated with a 0.057% rise in the enterprise carbon neutrality development index. Therefore, Hypothesis 1 is confirmed.

### 4.2. The Mediating Effect of Financing Incentives

To further examine the mediating effect of green fintech in promoting corporate carbon neutrality through financing incentives—specifically by alleviating financing constraints and reducing financial default distance—the results are reported in Table 5. In column (1) of Table 5, the results with financing constraints (*SA*) as the mediator show that the regression coefficient of green fintech is −0.001 and statistically significant at the 1% level. This indicates that a 1% increase in the green fintech development index is associated with a 0.001% reduction in financing constraints, suggesting that green fintech effectively mitigates corporate financing constraints. Thus, Hypothesis 2 is supported, confirming that green fintech provides financial support for corporate carbon-neutral development.

Column (2) of Table 5 presents the results with financial default distance (*FZ*) as the mediator. The regression coefficient of green fintech is −0.052 and remains significant at the 1% level, implying that a 1% increase in the green fintech development index corresponds to a 0.0517% decrease in financial default distance. This indicates that green fintech significantly reduces the financial default distance of enterprises, limits the accumulation of idle capital, and encourages greater investment in carbon neutrality projects. Consequently, Hypothesis 3 is verified.

### 4.3. The Regulatory Effect of Scale and Quality

To further examine how firm scale and audit quality moderate the impact of green fintech on corporate carbon neutrality, the study introduces two moderating variables: audit quality and firm size.

First, audit quality (*Audit*) is included to examine its moderating role in the relationship between green fintech and corporate carbon neutrality. Column (1) in Table 6 shows that the coefficient of green fintech is 0.044 and statistically significant at the 5% level. The interaction term between green fintech and audit quality is also positive (0.022) and significant at the 5% level. This suggests that when an enterprise is audited by one of the Big Four international accounting firms or a top-10 domestic audit firm, the positive effect of green fintech on corporate carbon neutrality is significantly enhanced. In other words, green fintech exerts a stronger promoting effect on carbon neutrality in firms with high audit quality. Thus, Hypothesis 4 is supported.

Second, firm size (*ES*) is introduced to test its moderating influence. Column (2) of Table 6 reports that the coefficient of green fintech alone is 0.021 but is not statistically significant even at the 10% level. However, the interaction term between green fintech and firm size is 0.052 and significant at the 1% level. This indicates that for firms with total assets above the sample median, the positive effect of green fintech on carbon neutrality is significantly stronger. Therefore, green fintech has a more pronounced promoting effect on the carbon neutrality development of large-scale enterprises. Hypothesis 5 is thereby verified.

### 4.4. Robustness Test

To address potential endogeneity, the one-period lagged explanatory variable is used as an instrumental variable. In the first stage, the F-statistic of the instrument exceeds the critical value and shows a positive correlation with green fintech at the 1% significance level, confirming that the instrument has strong explanatory power for the original endogenous variable. In the second stage, the *K-Paap rk LM* statistic indicates that the identification test is passed, and the Cragg–Donald Wald *F* statistic shows that the weak instrument test is passed, supporting the appropriateness of the selected instrumental variable. The regression results based on the instrumental variable approach yield a coefficient of 0.051 for green fintech, which remains positively significant at the 1% level. This confirms that green fintech does indeed have a positive effect on promoting corporate carbon neutrality development.

Existing literature suggests that a higher degree of digital transformation in firms is associated with a higher level of green innovation and a corresponding reduction in carbon emissions. The extent of digital transformation is measured by the combined frequency of five keywords—artificial intelligence, blockchain, cloud computing, big data, and digital technology applications—in corporate annual reports. The sum of these keyword frequencies constitutes the digital transformation (*DCG*) variable, which is then included as an additional fifth control variable. As reported in column (1) of Table 7, the regression coefficient of the green fintech development index on the enterprise carbon neutrality development index is 0.056 and remains significantly positive at the 1% level, indicating that the core findings are robust.

After excluding samples from the Xizang region, the results in column (2) of Table 7 show that the effect of the green fintech development index on the enterprise carbon neutrality development index remains largely unchanged, maintaining a significantly positive relationship at the 1% level. To account for the potential impact of the COVID-19 pandemic outbreak in 2019 on business operations, all enterprise samples from that year were also removed. The corresponding results, presented in column (3) of Table 7, indicate that even after excluding the 2019 samples, the significance of the green fintech development index on the enterprise carbon neutrality development index remains stable and statistically significant at the 1% level, further confirming the robustness of the findings.

## 5. Discussion and Conclusions

This study investigates the influence of green fintech on corporate carbon neutrality. Grounded in innovation economics and principal-agent theory, it formulates relevant hypotheses and develops two composite indicators: a green fintech development index and a corporate carbon neutrality development index. Utilizing a sample of Chinese A-share listed firms from 2014 to 2023, a two-way fixed-effects model is employed to assess the impact of green fintech on carbon neutrality. The analysis further explores the mediating role of financing incentives, the moderating effects of firm size and audit quality, and conducts comprehensive robustness tests.

The empirical findings indicate that baseline regressions confirm a positive relationship between green fintech and corporate carbon neutrality. Enhancements in green fintech development are associated with progress in both carbon neutrality and ESG performance. Mediation analysis reveals that green fintech advances carbon neutrality by alleviating corporate financing constraints and reducing financial default distance, thereby improving financing efficiency, easing funding pressures, and lowering the risk of financial distress. Moderation analysis demonstrates that audit quality and firm size significantly influence this relationship, with higher audit quality and larger firm size amplifying the positive impact of green fintech on carbon neutrality. Robustness tests, including endogeneity checks, alternative model specifications, and sample adjustments, consistently support these results. The green fintech development index remains statistically significant across all tests, underscoring the robustness of its role in promoting corporate carbon neutrality.

Based on the evidence, the following policy recommendations are proposed:

First, establish a shared green fintech service platform. Given its demonstrated efficacy in advancing corporate carbon neutrality, green fintech should be promoted through a multi-stakeholder framework, led by governments and involving financial institutions, technology firms, and research organizations. This platform should mobilize public and private capital to strengthen financial support for low-carbon innovation. By integrating corporate carbon-emission and credit data, it can help match firms with appropriate financing channels and reduce resource mismatches. Implementing a corporate carbon-account system—complete with a point-based mechanism to incentivize carbon-neutral behavior—would enable real-time progress tracking and facilitate tailored green financial services. The platform should prioritize openness and interoperability, linking with the national carbon-trading market and encouraging cross-regional cooperation to promote resource sharing and coordinated development.

Second, strengthen policy support for green fintech services. Achieving corporate carbon neutrality demands substantial financial investment. Policymakers should refine the top-level design and regulatory framework for green fintech to direct capital effectively toward carbon-neutrality projects. Enhanced risk-sharing mechanisms, along with clear incentives and penalties, will help unlock the inclusive potential of green fintech. Particular emphasis should be placed on supporting small and medium-sized enterprises (SMEs) and emerging green-technology firms, while stricter penalties are applied to traditional resource-intensive and high-pollution industries. The establishment of a dedicated carbon-neutrality fund could finance research and application of key technologies such as carbon substitution, mitigation, capture, and storage. Furthermore, green financial institutions should be encouraged to offer diversified financial products—including preferential loans and extended repayment terms—to enterprises with strong carbon-neutral performance.

Third, enhance the corporate carbon information disclosure system. Governments at all levels should establish comprehensive carbon-disclosure frameworks adapted to local development needs. This includes implementing stringent green-auditing standards, standardizing carbon-data structures and reporting formats, and adopting a unified carbon-accounting system. Companies should be required to disclose key ESG metrics—such as carbon-reduction volumes—supported by mandatory independent third-party verification to ensure accuracy and limit bias. Linking carbon performance with financial outcomes will bolster investor confidence and improve resource-allocation efficiency. Regulatory authorities must strengthen oversight of audit institutions by tightening qualification requirements, enhancing procedural supervision, and imposing stricter penalties for misconduct—thereby safeguarding auditor independence, professionalism, and overall market integrity. Additionally, implementation pathways should be tailored to disclosure quality and firm characteristics: Large firms with high audit quality should prioritize enterprise-wide green fintech (GFT) integration, align with Science-Based Targets initiative (SBTi) goals, and embed carbon pricing into capital budgeting; SMEs with high audit quality can focus on modular GFT solutions—such as supplier portals and energy dashboards—and access tailored green-financing instruments; Large firms with low audit quality should first invest in elevating assurance levels (e.g., from limited to reasonable assurance) and strengthen ESG data governance before scaling GFT; SMEs with low audit quality are advised to begin with foundational measures—including energy audits and basic IoT monitoring—and leverage FinTech microfinance or equipment-leasing models to improve efficiency.

Regarding the construction of the enterprise carbon-neutrality development index and the green fintech development index in this study, the selection of indicators has been constrained by data availability. Future research could improve precision by systematically collecting corporate carbon-emission data and GFT metrics from sources such as financial annual reports and ESG disclosures. Suggested future directions include: (1) Comparative studies of firms adopting GFT versus matched control groups before and after adoption, stratified by audit quality and firm size. (2) Event studies analyzing market reactions to GFT-related disclosures conditioned on the level of assurance. Such approaches would enable a more accurate and objective assessment of corporate progress toward carbon neutrality and access to green-financing support.

## Figures and Tables

**Table 1:** Enterprise carbon neutrality development index.

Variable	2014	2015	2016	2017	2018	2019	2020	2021	2022	2023
Min	8.78	8.77	9.03	10.12	10.14	9.76	11.89	11.71	13.19	12.18
Max	59.78	62.48	60.79	65.63	66.14	64.49	60.66	73.34	74.18	72.92
Max-Min	50.99	53.70	51.76	55.51	56.00	54.73	48.77	61.64	61.00	60.73
Std	8.87	8.93	9.29	9.39	9.61	9.94	9.56	10.42	10.92	11.48
Mean	22.56	23.63	24.34	25.09	26.03	26.64	26.56	28.87	30.17	32.36
N	2001	2194	2395	2811	2903	3077	3454	4001	3999	3994

**Table 2:** Trend of provincial green fintech development index.

Variable	2014	2015	2016	2017	2018	2019	2020	2021	2022	2023
Min	6.35	6.76	7.65	8.13	7.80	6.52	6.85	7.13	7.70	7.50
Max	42.78	44.77	48.38	54.51	50.30	49.04	50.60	53.60	52.76	54.24
Max-Min	36.43	38.01	40.73	46.38	42.50	42.52	43.75	46.47	45.06	46.74
Std	6.75	7.41	8.06	9.10	8.43	8.69	9.58	10.42	10.24	10.87
N	31	31	31	31	31	31	31	31	31	31

**Table 3:** Descriptive statistics.

Variable	N	Mean	Sd	Min	P50	Max
*Ecn*	30,653	27.2888	10.4662	8.7736	23.5493	74.1825
*GTF*	30,653	24.8152	12.5671	6.3468	21.8130	54.5063
*FZ*	30,653	5.2936	8.3304	−10.2675	3.2859	419.8183
*SA*	30,653	−3.8774	0.2780	−5.9802	−3.8755	−2.0850
*Audit*	30,653	0.6173	0.4861	0.0000	1.0000	1.0000
*Size*	30,653	22.3017	1.3403	18.3701	22.0830	28.6969
*Age*	30,653	2.9823	0.3078	1.3863	2.9957	4.8203
*ROA*	30,653	0.0386	0.0705	−1.5754	0.0388	0.7586
*TAGR*	30,653	0.1878	0.5510	−0.9290	0.0881	41.4625
*Balance*	30,653	0.7882	0.6281	0.0000	0.6258	4.0018

**Table 4:** Benchmark regression results.

Variable	(1)	(2)
	*Ecn*	*Ecn*
*GTF*	0.059 *** (0.020)	0.057 *** (0.020)
*Age*		3.471 ** (1.557)
*ROA*		1.592 ** (0.712)
*TAGR*		−0.044 (0.057)
*Balance*		−0.129 (0.212)
*_Cons*	22.147 *** (0.391)	12.823 *** (4.222)
Individual	Yes	Yes
Time	Yes	Yes
*R* ^2^	0.145	0.145
*N*	30,653	30,653

Note: The figures in parentheses are robust standard errors, ***, **, and * indicate significant at the 1%, 5%, and 10% levels.

**Table 5:** Mediation test results.

Variable	(1)	(2)
	*SA*	*FZ*
*GTF*	−0.001 *** (0.000)	−0.052 *** (0.017)
*Age*	−0.095 *** (0.021)	0.153 (1.450)
*ROA*	0.026 *** (0.006)	13.862 *** (1.016)
*TAGR*	−0.002 *** (0.001)	−0.700 *** (0.197)
*Balance*	0.010 *** (0.003)	0.164 (0.195)
*_Cons*	−3.419 *** (0.058)	5.887 (3.907)
Individual	Yes	Yes
Time	Yes	Yes
*R* ^2^	0.847	0.062
*N*	30,653	30,653

Note: The figures in parentheses are robust standard errors, ***, **, and * indicate significant at the 1%, 5%, and 10% levels.

**Table 6:** Results of the moderating effect test.

Variable	(1)	(2)
	*Ecn*	*Ecn*
*GTF*	0.044 ** (0.021)	0.021 (0.022)
*GTF × Audit*	0.022 ** (0.011)	
*Audit*	−0.220 (0.316)	
*GTF × ES*		0.052 *** (0.015)
*ES*		−0.268 (0.4422)
*Age*	3.461 ** (1.554)	3.189 ** (1.544)
*ROA*	1.530 ** (0.710)	1.370 * (0.715)
*TAGR*	−0.042 (0.057)	−0.064 (0.059)
*Balance*	−0.133 (0.212)	−0.169 (0.212)
*_Cons*	12.975 *** (4.224)	14.136 *** (4.186)
Individual	Yes	Yes
Time	Yes	Yes
*R* ^2^	0.146	0.147
*N*	30,653	30,653

Note: The figures in parentheses are robust standard errors, ***, **, and * indicate significant at the 1%, 5%, and 10% levels.

**Table 7:** Robustness test results.

Variable	(1)	(2)	(3)
	*Ecn*	*Ecn*	*Ecn*
*GTF*	0.056 *** (0.020)	0.054 *** (0.021)	0.057 *** (0.021)
*Age*	3.427 ** (1.557)	3.489 ** (1.566)	3.458 ** (1.578)
*ROA*	1.650 ** (0.712)	1.589 ** (0.715)	2.069 ** (0.828)
*TAGR*	−0.035 (0.057)	−0.044 (0.057)	−0.036 (0.0572)
*Balance*	−0.133 (0.213)	−0.128 (0.213)	−0.099 (0.225)
*DCG*	0.007 * (0.004)		
*_Cons*	12.919 *** (4.223)	12.838 *** (4.244)	12.818 *** (4.280)
Individual	Yes	Yes	Yes
Time	Yes	Yes	Yes
*R* ^2^	0.146	0.145	0.156
*N*	30,653	30,517	27,578

Note: The figures in parentheses are robust standard errors, ***, **, and * indicate significant at the 1%, 5%, and 10% levels.

## Data Availability

The data supporting the plots within this paper and other study findings are available from the corresponding author upon reasonable request.

## Appendix A

**Table A1:** The index system of the enterprise carbon neutrality development index.

First-Level Indicators	Second-Level Indicators	Third-Level Indicators	Efficacy	Proportion
Carbon offset	Environmental management system	Has it passed the ISO14000 certification	+	9.77%
		Has it passed the ISO9001 certification	+	9.64%
	The concept of emission reduction	Score of emission reduction concept	+	5.86%
	Green innovation investment	The proportion of R&D personnel	+	4.76%
		Proportion of R&D investment	+	1.99%
	Green innovation output	Green invention patent	+	16.58%
		Green utility model patent	+	13.87%
		Proportion of intangible assets	+	4.84%
Carbon emission reduction	Corporate carbon emissions	Carbon emission performance	+	2.00%
		Carbon dioxide emissions from enterprise coal	−	2.02%
		Carbon dioxide emissions from coke produced by enterprises	−	1.90%
		Enterprise crude oil carbon dioxide emissions	−	1.86%
		Enterprise gasoline carbon dioxide emissions	−	1.87%
		Enterprise kerosene carbon dioxide emissions	−	1.85%
		Enterprise diesel carbon dioxide emissions	−	1.88%
		Carbon dioxide emissions from enterprise fuel oil	−	1.87%
		Enterprise natural gas carbon dioxide emissions	−	1.97%
	Environmental accident	Environmental accident score	−	1.84%
	Carbon information disclosure	Environmental liability disclosure score	+	7.37%
		Environmental governance disclosure score	+	6.26%

Note: + indicates that the factor indicator has a positive impact on the comprehensive index, while − indicates that the factor indicator has a negative impact on the comprehensive index.

**Table A2:** The indicator system of the green fintech development index.

First-Level Indicator	Secondary Indicators	Third-Level Indicators	Efficacy	Specific Gravity
Resources	R&D personnel	R&D personnel/regional permanent resident population × 10,000	+	7.86%
	Research and development institution	The number of R&D institutions/the permanent resident population of the region	+	7.50%
	Financial resources	Regional financial industry added value/regional gross domestic product	+	4.31%
Funding	The intensity of financial appropriation	Expenditure on science and technology/general budget expenditure	+	5.99%
	Intensity of R&D investment	Intensity of R&D funding input	+	4.49%
	Scale of R&D investment	Total R&D expenditure	+	11.09%
Financing	Financial support intensity	Balance of loans from financial institutions/total R&D expenditure	+	12.61%
	Intensity of venture capital investment	Venture capital capital/GDP	+	15.07%
	Market financing scale	Market capitalization of technology-oriented listed companies	+	14.30%
Output	The transaction rate of the technology market	Technology market transaction volume/R&D expenditure	+	7.42%
	Paper output rate	The number of scientific and technological papers published/R&D expenditure	+	6.23%
	Output rate of new products	Sales revenue of new products in high-tech industries/main business income	+	3.12%

Note: + indicates that the factor indicator has a positive impact on the comprehensive index, while − indicates that the factor indicator has a negative impact on the comprehensive index.
